# Cognitive-behavioral intervention for math anxiety in childhood: a case report

**DOI:** 10.1590/1980-57642021dn15-020018

**Published:** 2021

**Authors:** Amanda Paola Lobo Guimarães, Vitor Geraldi Haase, Carmen Beatriz Neufeld

**Affiliations:** 1 Graduate Program in Neuroscience, Universidade Federal de Minas Gerais - Belo Horizonte, MG, Brazil.; 2 Psychology Department, Universidade Federal de Minas Gerais - Belo Horizonte, MG, Brazil.; 3 Psychology Department, Universidade de São Paulo - Ribeirão Preto, SP, Brazil.

**Keywords:** cognitive behavioral therapy, mathematics, anxiety, child, school, learning, terapia cognitivo-comportamental, matemática, ansiedade, criança, escola, aprendizagem

## Abstract

Math anxiety (MA) is a feeling of dread, tension and anxiety when dealing with math situations. Avoidance behavior prevents children from learning math, impairing their performance. Cognitive behavioral therapy is an approach with robust evidence of efficacy in treating anxiety disorders. The Coping Cat, a protocol for treating anxiety disorders, inspired the MA intervention reported here. The participant was N. L., an 11-year-old female displaying math-related and math-unrelated anxiety symptoms. Outcome measures included arithmetic performance, and self-reports of general positive and negative affect and math-related self-perceived performance, attitude, unhappiness and anxiety. The therapeutic approach included psychoeducation, relaxation, diaphragmatic breathing, cognitive restructuring, problem-solving training and graded exposure. After 12 individual intervention sessions, there was an improvement in negative and positive affect, as well as an improvement in self-perceived performance and a decrease in MA.

## RESUMO

A ansiedade matemática (AM) pode ser definida como sentimento de tensão e ansiedade ao se manipular estímulos numéricos. O comportamento de esquiva, típico da ansiedade, impede que a criança treine matemática, prejudicando seu desempenho. A terapia cognitivo-comportamental é uma abordagem com evidências robustas de eficácia no tratamento da ansiedade e, ao longo do tempo, diversos protocolos de intervenção foram desenvolvidos para esse distúrbio. Um protocolo infantil conhecido por sua eficácia é o *Coping Cat*, o qual inspirou a elaboração da intervenção para AM do presente estudo. N. L., 11 anos, foi classificada como superior em uma escala de AM. Após 12 sessões de intervenção individuais, ela alcançou escores médios na mesma escala. As técnicas utilizadas foram psicoeducação, respiração diafragmática, relaxamento, reestruturação cognitiva, treinamento de resolução de problemas e exposição.

## INTRODUCTION

Around 17% of the population have high levels of math anxiety (MA).^1^ MA is a form of phobia, in which manifestations include cognitive (negative attitudes, worrisome rumination, feelings of helplessness, low self-esteem and self-efficacy, etc.), affective (dysphoria), behavioral (avoidance, hurry-up to finish math tasks, etc.), and physiological (sweating, trembling, high pulse rate, etc.) symptoms.^2^ MA is both a cause and a consequence of low math performance. MA interferes with processing during math activities and avoidance behaviors reduce learning opportunities. MA and low math performance are related to outcomes such as bullying, school dropout, avoidance of advanced courses in mathematics and low-paying careers.^3^

MA is not recognized as a nosological entity and there are no gold standard external criteria of diagnosis, and as a consequence, interventions for MA are under-researched. Bicer et al.^4^ in their meta-analysis showed that the overall effect size of cognitive-behavior therapy (CBT) in reducing students’ MA was moderate to strong (g=-0.76 with SD=0.04).

Early interventions are important to avoid the negative effects of MA. Accordingly, to fill this gap, the present study aimed at producing preliminary scientific evidence of the effectiveness of CBT for MA in children, using a single-case study.

## METHODS

### Participant

The middle-class girl was 11 years old, had normal intelligence and was in the 5th grade in a state-run school. Achievement in math was above PR75 and below PR25 in spelling. Speech and language were delayed. She showed symptoms of anxiety in daily situations, especially when reading in public and taking math tests. Participation was conditioned to written (parents) and oral (child) informed consent. The research project was previously approved by the local ethics board (COEP-UFMG, 01934818.4.0000.5149).

### Intervention

The Coping Cat program^5^ was adopted because of its efficacy in the treatment of anxiety in 7- to 14-year-olds.^6^ The intervention taught to recognize anxiety, manage perceived anxiety levels and master challenging anxiogenic tasks. The program consisted of 12 weekly sessions lasting 50 minutes:

- Sessions 1 and 2. Rapport was established through non-directed conversation and through a "personal facts" game, familiarizing the child with the environment and the therapist. Toys were in sight, and it was suggested that the two could engage in games at the end of the session. The child received guidance about the program, being reinforced by participation.
- Sessions 3. Management of somatic reactions such as tremor, heart racing, dry throat and “butterflies” in her stomach. Setting-up a hierarchy of anxiety situations, from the lowest the highest. Identification of dysfunctional thoughts: "I'm going to make a mistake"; I'm going to stutter"; "I should have studied more"; I'm going to get a bad grade"; "the test will be difficult"; "I'll forget everything"; "I'm going to get a reprimand".
- Session 4. Relaxation training.
- Session 5. Role of thoughts. Dysfunctional thought: Constant feelings of not having studied enough and fear of failing the exams in spite of good grades. Considering the evidence against her thoughts of failing the tests and concluding that if she studied the day before the exam, as she usually did, she would probably do as well as usual, not needing to study during school recess on the day of the test. Learning alternative, more adaptive thoughts: "If I study for the test, I’ll probably do well"; "I'm still learning, so it's normal to make mistakes"; "If I try to solve the math problem, I have a better chance of getting it right than if I do not even try"; "If I do not know something, the teacher or my mother can help me".
- Session 6. Problem-solving training. Difficulties doing the math homework with feelings of not knowing how and getting anxious. Learned adaptive thoughts: "It is normal to make mistakes when one is learning" "Trying to solve the question the best way possible, even not being sure if the result is correct"; and "Asking her mother and her teacher for help when she cannot solve a question".
- Sessions 8 to 12. Hierarchical exposure to the increasingly anxiety-eliciting situations previously identified until a 50% reduction in the subjective self-report of anxiety was obtained.

### Instruments

The following psychological dimensions were assessed: a) intelligence using Raven's Coloured Progressive Matrices;^7^ b) single-digit calculation applying Simple Calculation Task - SCT;^8^ c) well-being through Positive and Negative Affect Scale - PNAS;^9^ d) the Math Anxiety Questionnaire (MAQ), an instrument used to evaluate children’s perceptions of their own skills and feelings, when performing math activities or having difficulties in performing them, was the main outcome measure,^10^ ^,^ ^11^ where MAQ is composed of four subscales: Self-perceived Performance; Attitude; Unhappiness; and Anxiety; and e) self-rated anxiety before and after the exposure sessions was the secondary outcome measure.

### Procedures

A within subjects pre- and post-test design was adopted. Potential improvements produced by the intervention were measured by single-digit calculations, MAQ and PNAS. Intervention sessions took place three months apart, between pre- and post-test. A six months follow-up was applied.

### Statistical analyses

McNemar tests were conducted to compare the pre- and post-test scores with a significance level of p<05.

## RESULTS

The intervention was successful in modifying both dysfunctional cognitions, assessed by the MAQ, and reducing anxiety during exposure. Pre- and post-test comparisons are shown in Table 1. At pre-test, the girl performed above PR75 in all MAQ subscales. In the pre-test and follow-up, her responses were situated below PR25, with the exception of Attitudes, which persisted high. Comparatively to the pre-test, MA symptoms were reduced both in the post-test as in the follow-up. However, results were statistically significant only at the post-test for the Self-perceived Performance and Anxiety subscales of MAQ. Positive affect in the PNAS significantly increased and negative affect significantly decreased both in the post-test as in the follow-up. No change occurred in the performance in single-digit calculations.

**Table 1.** Scores on pre- and post-test for the Simple Calculation Task, Positive and Negative Affect Scale and Math Anxiety Questionnaire.

Test	Measured construct	Pretest		Posttest		Follow-up		Pre-Posttest	Pretest-Follow-up
		Raw score	Z score	Raw score	Z score	Raw score	Z score	chi-square	chi-square
SCT	Addition	24	0,03	22	-0,66	26	0,73	0,5	0,5
	Subtraction	18	0,18	17	-0,02	15	-0,42	0,5	1,33
	Multiplication	17	0,01	18	0,16	17	0,01	0	0
PNAS	Negative affect	27	-0,37	18	-1,19	17	-1,28	7,11**	8.10**
	Positive affect	43	-2,40	64	-0,24	61	-0,55	19,04***	16.05**
MAQ	Self-perceived	21	1,90	15	0,38	18	1,14	4,16*	1.33
	Attitude	20	0,93	17	0,28	21	1,14	1,33	0.00
	Unhappiness	28	2,70	23	1,58	23	1,58	3,2	3.20
	Anxiety	27	1,91	19	0,21	23	1,06	6,12*	2.25

Pre-Posttest: comparison between pre and posttest. Pretest-Follow-up: comparison between pretest and follow-up; *p<0.05, **p<0.01, ***p<0.001.

Improvement in MA symptoms was also observed through self-ratings in the exposure sessions (Table 2). The situations were worked out according to the child's subjective anxiety score, starting with the ones that generated the least anxiety to the ones that generated the most.

**Table 2.** Self-reported subjective anxiety in the exposure sessions

Session	Situation	Anxiety at the beginning	Anxiety at the end
8^th^	Read in front of the class	5	1,5
9^th^	Math test	5	2
10^th^	Present a math work before class	7	3
11^th^	Go to the board to solve a calculation	10	5
12^th^	Not being able to conclude math homework	10	5,5

Note: the reported anxiety score varied on a scale from zero to 10.

According to the parents, the child showed a decrease in her anxiety before the math tests. They noticed improvement mainly in the well-being of the child, who seemed “happier and more interested in interacting at home and at school”. In addition, the child reported to the therapist that "now, before I start to worry, I try to solve it again and if I can't, I ask the teacher or my mother" when she was doing an exercise.

## DISCUSSION

In this study, we investigated the outcome of a MA cognitive-behavioral intervention in a pre- and post-test single-case study of a school child. There was an increase in general positive affect and decrease in general negative affect. MA symptom intensity decreased from high to medium. Arithmetic performance did not change.

Treating MA in a child is an original aspect of the present study. Most studies have focused on the treatment of MA in adolescents and adults.^12^ ^,^ ^13^ ^,^ ^14^ The results of the present study suggest that a comprehensive CBT program may be effective in treating symptoms of MA in children.

No effects of the intervention were observed on math achievement, in contrast with results obtained with some^12^ ^,^ ^14^ but not all studies with adolescents and adults.^13^ Although MA is observed in children, it is not always related to poor performance.^10^ ^,^ ^11^ ^,^ ^15^ ^,^ ^16^ MA generally decreases math achievement in the short- and long-run. Math learning difficulties are a risk factor for MA.^17^ However, not all children with math difficulties develop MA symptoms. Math achievement in the participant of the previous study was average. Improvements in math achievement are generally observed in individuals with low performance.^12^ ^,^ ^14^ According to Dowker et al.,^18^ another possibility is that the MAQ used in the present study places more emphasis on the cognitive (“worry”) than on the affective aspects of mathematics. This is in line with observations of Orbach and coworkers^19^ ^,^ ^20^ that MA can be a bidimensional construct. An affect-loaded state dimension is more strongly associated with performance and thus more susceptible to treatment than a cognitive-loaded trait dimension.

The distinction between state and trait MA^19^ ^,^ ^20^ could also be relevant for the interpretation of the treatment effects on the different MAQ subscales. Self-reports indicated significant improvements in both affective components of MA on the order of more than one standard deviation. Similar significant improvement was observed for self-perceived performance. A non-significant 0.6 standard deviation reduction in negative attitudes toward math was also observed. This suggests a brief intervention may not be so effective in the reduction of long-standing negative attitudes toward math.

MA anxiety could then be considered the outcome of an anxiety-diathesis interacting with negative experiences with math.^21^ ^,^ ^22^ Our results then suggest that CBT could be an effective intervention for MA symptoms in school-age children. Furthermore, systematic and more powerful studies are required to test this hypothesis.
